# The evaluation of monoclonal gammopathy of renal significance: a consensus report of the International Kidney and Monoclonal Gammopathy Research Group

**DOI:** 10.1038/s41581-018-0077-4

**Published:** 2018-12-03

**Authors:** Nelson Leung, Frank Bridoux, Vecihi Batuman, Aristeidis Chaidos, Paul Cockwell, Vivette D. D’Agati, Angela Dispenzieri, Fernando C. Fervenza, Jean-Paul Fermand, Simon Gibbs, Julian D. Gillmore, Guillermo A. Herrera, Arnaud Jaccard, Dragan Jevremovic, Efstathios Kastritis, Vishal Kukreti, Robert A. Kyle, Helen J. Lachmann, Christopher P. Larsen, Heinz Ludwig, Glen S. Markowitz, Giampaolo Merlini, Peter Mollee, Maria M. Picken, Vincent S. Rajkumar, Virginie Royal, Paul W. Sanders, Sanjeev Sethi, Christopher P. Venner, Peter M. Voorhees, Ashutosh D. Wechalekar, Brendan M. Weiss, Samih H. Nasr

**Affiliations:** 1 Division of Nephrology, Hematology, Department of Laboratory Medicine and Pathology, Mayo Clinic, Rochester, MN USA; 2 Department of Nephrology, Centre Hospitalier Universitaire et Université de Poitiers, Poitiers, France; CNRS UMR7276, Limoges, France; and Centre de Référence Amylose AL et Autres Maladies par Dépôt d’Immunoglobulines Monoclonales, Poitiers, France; 3 Veterans Administration Medical Center, New Orleans, LA, USA and Tulane University Medical School, Tulane, LA USA; 4 Centre for Haematology, Department of Medicine, Imperial College London and Imperial College Healthcare NHS Trust, Hammersmith Hospital, London, UK; 5 Department of Nephrology, Renal Medicine — University Hospitals Birmingham NHS Foundation Trust, Queen Elizabeth Hospital, Birmingham, UK; 6 Department of Pathology, Renal Pathology Laboratory, Columbia University, College of Physicians and Surgeons, New York, NY USA; 7 Department of Haematology and Immunology, University Hospital St Louis, Paris, France; 8 The Victorian and Tasmanian Amyloidosis Service, Department of Haematology, Monash Univerity Easter Health Clinical School, Melbourne, Victoria Australia; 9 National Amyloidosis Centre, Centre for Amyloidosis and Acute Phase Proteins, Division of Medicine, Royal Free Campus, University College London, London, UK; 10 Department of Pathology and Translational Pathobiology, Louisiana State University Health Sciences Center, Shreveport, LA USA; 11 Service d’Hématologie et de Thérapie Cellulaire, Centre de Référence des Amyloses Primitives et des Autres Maladies par Dépôts d’Immunoglobuline, CHU Limoges, Limoges, France; 12 Department of Clinical Therapeutics, School of Medicine National and Kapodistrian University of Athens Alexandra Hospital, Athens, Greece; 13 University Health Network, Princess Margaret Cancer Centre, Toronto, Canada; 14 Arkana Laboratories, Little Rock, AR USA; 15 Wilhelminen Cancer Research Institute, Wilhelminenspital, Vienna, Austria; 16 Amyloidosis Research and Treatment Center, IRCCS Policlinico San Matteo, University of Pavia, Pavia, Italy; 17 Haematology Department, Princess Alexandra Hospital and School of Medicine, University of Queensland, Brisbane, Australia; 18 Department of Pathology, Loyola University Medical Center, Maywood, IL USA; 19 Department of Pathology, Hôpital Maisonneuve-Rosemont, Université de Montreal, Montreal, Quebec, Canada; 20 Department of Medicine, University of Alabama at Birmingham and Department of Veterans Affairs Medical Center, Birmingham, AL USA; 21 Cross Cancer Institute, University of Alberta, Edmonton, Alberta Canada; 22 Department of Hematologic Oncology and Blood Disorders, Levine Cancer Institute, Atrium System, Charlotte, NC USA; 23 Abramson Cancer Center, University of Pennsylvania, Perelman School of Medicine, Philadelphia, PA USA

**Keywords:** Renal cancer, Oncogenesis, Nephritis, Kidney, Pathology

## Abstract

The term monoclonal gammopathy of renal significance (MGRS) was introduced by the International Kidney and Monoclonal Gammopathy Research Group (IKMG) in 2012. The IKMG met in April 2017 to refine the definition of MGRS and to update the diagnostic criteria for MGRS-related diseases. Accordingly, in this Expert Consensus Document, the IKMG redefines MGRS as a clonal proliferative disorder that produces a nephrotoxic monoclonal immunoglobulin and does not meet previously defined haematological criteria for treatment of a specific malignancy. The diagnosis of MGRS-related disease is established by kidney biopsy and immunofluorescence studies to identify the monotypic immunoglobulin deposits (although these deposits are minimal in patients with either C3 glomerulopathy or thrombotic microangiopathy). Accordingly, the IKMG recommends a kidney biopsy in patients suspected of having MGRS to maximize the chance of correct diagnosis. Serum and urine protein electrophoresis and immunofixation, as well as analyses of serum free light chains, should also be performed to identify the monoclonal immunoglobulin, which helps to establish the diagnosis of MGRS and might also be useful for assessing responses to treatment. Finally, bone marrow aspiration and biopsy should be conducted to identify the lymphoproliferative clone. Flow cytometry can be helpful in identifying small clones. Additional genetic tests and fluorescent in situ hybridization studies are helpful for clonal identification and for generating treatment recommendations. Treatment of MGRS was not addressed at the 2017 IKMG meeting; consequently, this Expert Consensus Document does not include any recommendations for the treatment of patients with MGRS.

## Abstract (web summary)

This Expert Consensus Document from the International Kidney and Monoclonal Gammopathy Research Group includes an updated definition of monoclonal gammopathy of renal significance (MGRS) and recommendations for the use of kidney biopsy and other modalities for evaluating suspected MGRS

## Introduction

The concept of monoclonal gammopathy of undetermined significance (MGUS) was first introduced in 1978 by Robert Kyle^1^. This premalignant condition is characterized by the presence of a serum monoclonal immunoglobulin <30 g/l and <10% monoclonal bone marrow plasma cells in a patient who does not have any organ damage attributable to the monoclonal immunoglobulin. Conversion of MGUS to malignancy, which mandates the initiation of appropriate treatment, is indicated by the development of disease-specific features. For example, conversion to multiple myeloma (MM) is indicated by the occurrence of one or more myeloma-defining events, such as hypercalcaemia, renal impairment, anaemia, lytic bone lesions or an event suggestive of impending myeloma (such as a serum involved:uninvolved free light-chain ratio >100, >60% bone marrow plasma cells or ≥1 bone lesions on MRI)^2^. Progression to Waldenström macroglobulinaemia (WM) is indicated by the development of anaemia, thrombocytopenia, bulky adenopathy or organomegaly, blood hyperviscosity, severe neuropathy, amyloidosis, cryoglobulinaemia, cold agglutinin disease or malignant transformation^3^. Similarly, treatment for chronic lymphocytic leukaemia (CLL) is initiated when a patient with MGUS develops cytopenias, progressive or symptomatic lymphadenopathy, organomegaly or constitutional symptoms^4^. Patients with MGUS who do not yet exhibit any of these disease-specific features do not require treatment but should undergo careful monitoring^2,5–9^.

The kidney is commonly involved in these haematological malignancies. Light-chain cast nephropathy is now considered a myeloma-defining event, although it is not exclusive to MM^10^. In addition to cryoglobulinaemic glomerulonephritis, a variety of other kidney diseases have been observed in patients with WM, including immunoglobulin light-chain (AL) amyloidosis, monoclonal immunoglobulin deposition disease (MIDD), light-chain proximal tubulopathy (LCPT) and, on rare occasions, cast nephropathy^11–13^. Similar renal lesions have also been described in patients with CLL^14^. Importantly, however, these kidney diseases have also been described in patients with a low clonal burden (defined as monoclonal immunoglobulin <30 g/l and <10% monoclonal bone marrow plasma cells) who therefore do not meet the diagnostic criteria for MM or other malignancies. In the past, these patients were categorized as having ‘idiopathic’ light-chain disposition disease or ‘primary’ amyloidosis^15,16^. The fact that these kidney lesions have been replicated in animal models by Bence Jones protein injections alone further supports the notion that the presence of MM is not required^17,18^. For this reason, the International Myeloma Working Group does not consider patients with plasma cell dyscrasia and kidney diseases other than cast nephropathy to have MM unless they also exhibit other myeloma-defining events^2^.

The occurrence of kidney diseases associated with a monoclonal gammopathy in the absence of symptomatic MM, WM or CLL is increasingly recognized^10^. Most of these patients have a small, low-grade clonal disorder that is similar to MGUS, although (unlike MGUS) these clones do cause vital organ damage — including neuropathy, cardiomyopathy, hepatic dysfunction and dermopathy — mediated by the monoclonal immunoglobulin^2,19,20^. The clonal aetiology of these diseases results in clinical features that differ from those of non-monoclonal gammopathies, such as membranous nephropathy or IgA nephropathy. For example, monoclonal immunoglobulin-related diseases tend to be progressive and are unlikely to undergo spontaneous remission^21–25^. Monoclonal immunoglobulin-related diseases also show higher rates of recurrence after kidney transplantation (often >80%) than their non-monoclonal counterparts^26–28^. Monoclonal diseases are poorly responsive to conventional immunosuppression and instead require clone-directed therapy^25,29–32^.

Increasing recognition of the relationship between monoclonal gammopathies and kidney disease generated the need for more-accurate classification of these disorders, which were previously often misdiagnosed or categorized as unclassifiable by existing disease criteria. Moreover, as the use of cytotoxic therapy is typically limited to patients with MM, WM or CLL, patients with monoclonal gammopathy-related kidney diseases (who do not meet the criteria for these malignancies) were left without access to these essential drugs^2^. Accordingly, a series of meetings was organized by the International Kidney and Monoclonal Gammopathy Research Group (IKMG) with the aim of designating these clonal disorders as pathologies distinct from MGUS and thereby enabling government agencies to allocate resources for their treatment. In 2012, the IKMG introduced the term monoclonal gammopathy of renal significance (MGRS) to describe haematological conditions that produce a monoclonal immunoglobulin associated with kidney injury^33^. Since then, the IKMG has published recommendations for the treatment of MGRS^34^ and a classification scheme for MGRS-related renal lesions^35^. The IKMG met again in New Orleans, Louisiana, United States, in 2017 to update the classification of MGRS-associated renal lesions as well as to refine the definition of MGRS. The present Expert Consensus Document is derived from these discussions, which occurred both face to face and in e-mail exchanges that incorporated the views of IKMG members who could not be present. The treatment of MGRS was not discussed at the meeting; therefore, this topic is not updated in this consensus document.

## Updated definition of MGRS

The original definition of MGRS included all small B cell clones that produced a toxic monoclonal protein^33^. Although this definition was based on the dangerous small B cell clones concept^19^, the nature of the clonal disease was not well defined. Specific questions arose regarding whether patients with smouldering (indolent) MM (SMM) or smouldering (indolent) WM (SWM) should be considered to have MGRS. Similar confusion existed with regard to the inclusion of patients with low-grade CLL or lymphoma, who do have a diagnosis of a malignancy but do not require treatment. The new definition includes all B cell and plasma cell clonal proliferative disorders that do not require immediate treatment for the clonal disease. In addition, the toxic monoclonal protein is now specified to be a nephrotoxic monoclonal immunoglobulin^33^.

The new IKMG consensus definition of MGRS (Box 1) includes all B cell or plasma cell proliferative disorders (such as SMM, SWM and monoclonal B cell lymphocytosis (MBL; a diagnosis that is the equivalent of MGUS for clones of the CLL lineage)) that produce a nephrotoxic monoclonal immunoglobulin^1,4,36,37^. Low-grade CLL and low-grade B cell non-Hodgkin lymphomas, such as marginal zone lymphoma, mantle-cell lymphoma or mucosa-associated lymphoid tissue (MALT) lymphoma are also considered to be MGRS when they are associated with renal lesions^38–41^ (Table 1). These low-grade proliferative disorders would be classified as MGUS, and affected patients would be monitored for progression but not offered treatment if not for the renal injury^19^. In patients who develop renal lesions as a result of the monoclonal immunoglobulin, therapeutic intervention is required to prevent further damage resulting in end-stage renal disease. Accordingly, the diagnosis of MGRS does not require the presence of any defining features of an overt lymphoplasmacytic malignancy and particularly not the presence of any myeloma-defining event.

**Table 1:** Characteristics of clonal B cell and plasma cell proliferative disorders

Disease	Clone	Bone marrow involvement	Immunoglobulin	M-spike	Organ damage and/or involvement
MGUS	Any	<10%	Any	<30 g/l	None
Smouldering MM^a^	Plasma cell	10–60%	Any	≥30 g/l	None
MM^a^	Plasma cell	≥10%	Any	≥30 g/l	SLiM CRAB: 60% bone marrow plasma cells, involved:uninvolved free light-chain ratio >100, >1 bone lesion on MRI, hypercalcaemia, renal impairment, anaemia and lytic bone lesions
Smouldering WM^a^	Lymphoplasmacytic lymphoma clone^b^	≥10%	IgM	≥30 g/l	Absent
WM^a^	Lymphoplasmacytic lymphoma clone^b^	≥10%	IgM	≥30 g/l	Anaemia, hyperviscosity, constitutional symptoms, bulky lymphadenopathy, hepatosplenomegaly and neuropathy
MBL	B-cell clone^c^	Peripheral B-cell count <5 × 10^9^/l	Any	Any	Absence of lymph node involvement
CLL	B-cell clone^c^	Peripheral B-cell count >5 × 10^9^/l	Any	Any	Adenopathy, anaemia and thrombocytopenia
Other B cell lymphoproliferative disorders	Pan B-cell markers (CD19^+^CD20^+^CD79^+^CD22^+^PAX5^+^)	Presence or absence	Any	Any	Adenopathy and splenomegaly

CLL, chronic lymphocytic leukaemia; MBL, monoclonal B cell lymphocytosis; MGUS, monoclonal gammopathy of undetermined significance; MM, multiple myeloma; SLiM CRAB, symptomatic, light chains, MRI, high calcium, renal dysfunction, anaemia, and bony lytic lesions; WM, Waldenström macroglobulinaemia. ^a^Either bone marrow involvement or an M-spike above these thresholds is sufficient for the diagnosis. ^b^Typically, B cells are surface IgM^+^CD5^−^CD10^−^CD11c^−^CD19^+^CD20^+^CD22^+^CD23^−^CD25^+^CD27^+^FMC7^+^CD103^−^CD138^−^ with a plasmacytic component that is CD138^+^CD38^+^CD19^+^CD45^+^CD56^−^. ^c^CD5^+^CD19^+^CD23^+^surface immunoglobulin^+^CD20^dim^.

Once the haematological condition progresses to overt MM, WM, advanced stage CLL or malignant lymphoma (as defined by their respective established disease criteria), these diseases are no longer considered MGRS and affected patients are managed according to disease-specific protocols.

Box 1 Updated definition of MGRS The following consensus view of monoclonal gammopathy of renal significance (MGRS) has emerged.

The term MGRS applies specifically to any B cell or plasma cell clonal lymphoproliferation with both of the following characteristics:

- One or more kidney lesions that are related to the produced monoclonal immunoglobulin
- The underlying B cell or plasma cell clone does not cause tumour complications or meet any current haematological criteria for specific therapy

## Updated classification system

### Terminology

**Fig. 1:**
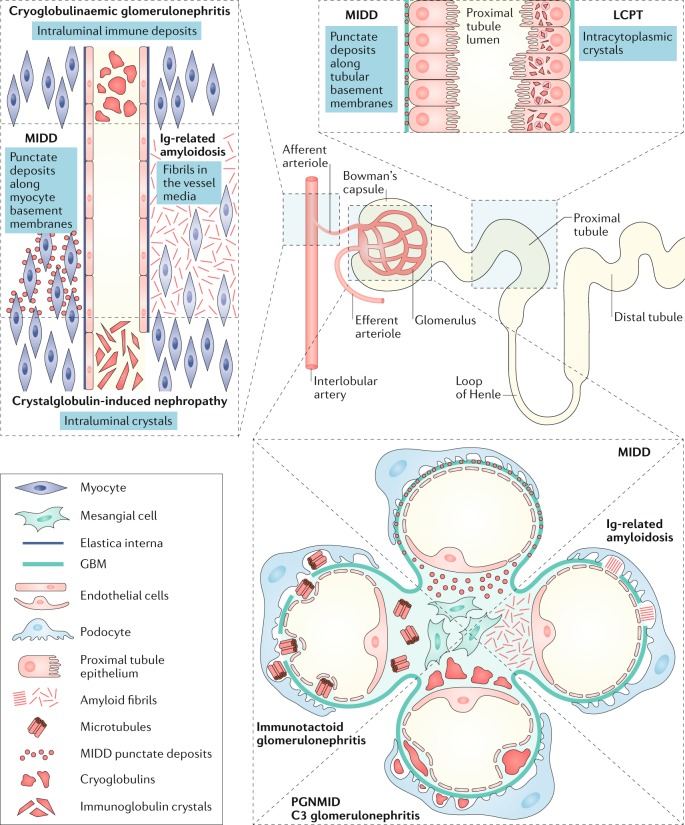
Localization of MGRS-associated renal lesions. Monoclonal gammopathy of renal significance (MGRS)-associated lesions can involve one or more renal compartments. In immunotactoid glomerulonephritis, C3 glomerulopathy and proliferative glomerulonephritis with monoclonal immunoglobulin deposits (PGNMID), MGRS-associated lesions involve only the glomeruli, whereas in light-chain proximal tubulopathy (LCPT), MGRS-associated lesions involve only the proximal tubules. MGRS-associated lesions in cryoglobulinaemic glomerulonephritis mainly involve the glomeruli but can occasionally affect blood vessels in the form of intravascular cryoglobulin thrombi or endovasculitis. Immunoglobulin-related amyloidosis and monoclonal immunoglobulin deposition disease (MIDD) usually affect all renal compartments, including glomeruli, vessels and the tubulointerstitium. GBM, glomerular basement membrane.

A variety of renal diseases have now been described in association with MGRS^35^ (Fig. 1). The IKMG recommends that these should be referred to as MGRS-associated lesions, conditions or disorders. Thus, for instance, classic AL amyloidosis might be considered an MGRS-associated condition when renal involvement is present. By contrast, the term MM-associated AL amyloidosis would be used when the same renal condition is associated with a symptomatic high tumour mass accompanied by at least one classic myeloma-defining event.

The type of renal lesion is governed by the innate structural characteristics and physicochemical properties of the monoclonal immunoglobulin rather than by the features of the clone that produced it^17^. Except for C3 glomerulopathy and thrombotic microangiopathy, which are not associated with renal deposition of monoclonal immunoglobulin, most MGRS-associated lesions are caused by the deposition of entire or parts of the monoclonal immunoglobulins or of various products of aggregation. Monoclonal immunoglobulin deposits in the kidney are generally restricted to immunoglobulin light chains (except in diseases that show a monoclonal immunoglobulin heavy-chain restriction, such as heavy-chain deposition disease or immunoglobulin heavy-chain amyloidosis). For example, in AL amyloidosis, the renal deposits are composed of only a single light chain^35^. In conditions where the entire immunoglobin is deposited, demonstration of both heavy-chain and light-chain restrictions are required to provide evidence of monoclonality.

**Fig. 2:**
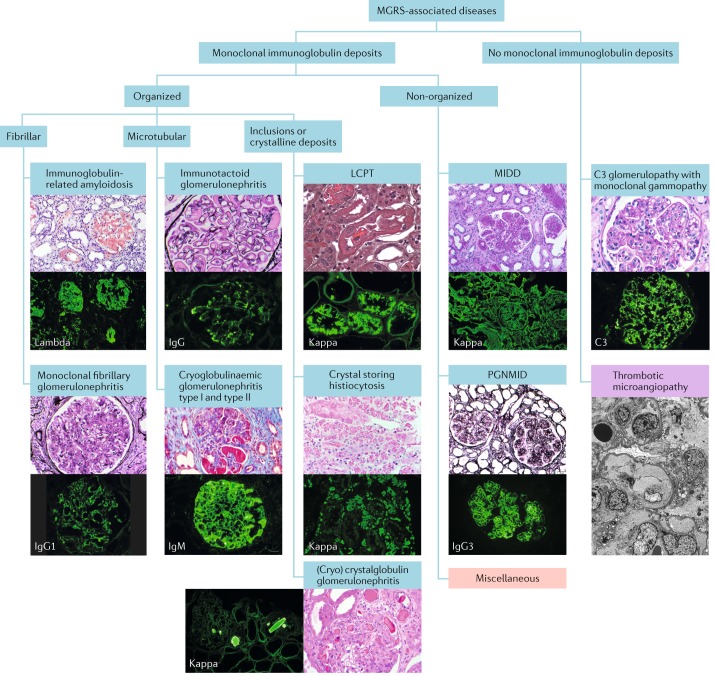
Categorization of MGRS-associated renal lesions. Monoclonal gammopathy of renal significance (MGRS)-associated renal lesions (blue boxes) are initially separated by the presence or absence of monoclonal immunoglobulin deposits in kidney biopsy samples. They are further subcategorized by the ultrastructural characteristics of the deposits into organized and non-organized. Organized deposits are further subdivided into fibrillar, microtubular and inclusions or crystalline categories. Images of typical histological sections stained with haematoxylin and eosin (H&E), periodic acid–Schiff or Masson trichrome stain and Congo red (top) are paired with immunofluorescence studies of frozen tissue sections (bottom) to reveal the specific immunoglobulin species. Pink box: the miscellaneous category represents polyclonal glomerulopathies that sometimes present with monoclonal immunoglobulin deposits, such as monotypic membranous nephropathy and monotypic anti-glomerular basement membrane disease. Purple box: thrombotic microangiopathy currently has a provisional status as an MGRS-associated lesion pending further evidence. Because this lesion has no immunoglobulin deposits and is best identified by electron microscopy, the immunofluorescence and H&E stained sections were replaced by an electron micrograph. LCPT, light-chain proximal tubulopathy; MIDD, monoclonal immunoglobulin deposition disease; PGNMID, proliferative glomerulonephritis and monoclonal immunoglobulin deposits.

The classification scheme proposed in 2017 by the IKMG for MGRS-associated lesions (Fig. 2) is based on the findings of immunofluorescence studies and the ultrastructural appearance of the deposits on electron microscopy. However, electron microscopy is not universally available, even in industrialized countries; consequently, the IKMG classification encourages but does not mandate the use of electron microscopy in the assessment of MGRS-associated disorders. By contrast, light microscopy and immunofluorescence studies with a full panel of antibodies are invariably required. The renal deposits are initially categorized as organized, non-organized and non-immunoglobulin. At the 2017 IKMG meeting in New Orleans, two additional subcategories were added to the non-organized and non-immunoglobulin categories of the classification scheme^35^. Thrombotic microangiopathy associated with monoclonal gammopathy was provisionally added as a subcategory of non-immunoglobulin deposits^42^, and a miscellaneous subcategory was added to the non-organized deposit category, which applies to pathological entities that are ultrastructurally similar to a non-monoclonal-immunoglobulin-related disease but are only sometimes associated with a monoclonal gammopathy. The MGRS-associated disorders included in this classification are discussed in more detail below.

### Lesions with organized deposits

**Fig. 3:**
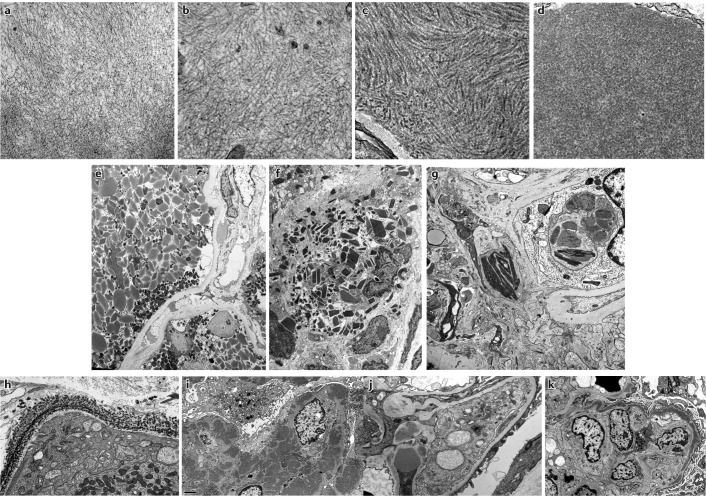
Ultrastructural appearance of MGRS-associated lesions. Top row: electron microscopy images showing fibrillar or microtubular deposits. **a** | Small randomly oriented fibrils of mean thickness 10 nm in a patient with immunoglobulin light-chain-κ amyloidosis (original magnification ×49,000). **b** | Randomly oriented fibrils with mean thickness of 15 nm in a patient with fibrillary glomerulonephritis (original magnification ×52,000). **c** | Deposits composed of microtubules with hollow centres organized in parallel arrays and with a mean thickness of 26 nm in a patient with immunotactoid glomerulopathy (original magnification ×49,500). **d** | Focal deposits composed of short microtubules with hollow centres with a mean thickness of 29 nm in a patient with cryoglobulinaemic glomerulonephritis (original magnification ×40,000). Centre row: electron microscopy images showing crystals or inclusions. **e** | Proximal tubular cells filled with moderately electron-dense, light-chain crystals that have rod and rhomboid shapes in a patient with crystalline light-chain proximal tubulopathy. The crystals are predominantly free within the cytoplasm, not membrane bound (original magnification ×2,700). **f** | Numerous light-chain crystals with rod, rectangle or rhomboid shapes within the cytoplasm of interstitial infiltrating histiocytes in a patient with crystal-storing histiocytosis (original magnification ×4,200). **g** | Needle-shaped, electron-dense crystals in the mesangium and within phagolysosomes of infiltrating inflammatory cells in a patient with cryocrystalglobulinaemia (original magnification ×9,300). The crystals showed monotypic staining for IgG and κ light chains on pronase immunofluorescence. Bottom row: electron microscopy images showing non-organized deposits. **h** | Finely granular, highly electron-dense deposits along a tubular basement membrane in a patient with light-chain deposition disease (original magnification ×15,000). **i** | Large, discrete (mesangial, subendothelial and subepithelial) granular, electron-dense deposits in a patient with proliferative glomerulonephritis with monoclonal immunoglobulin deposits (original magnification ×6,000). **j** | Mesangial deposits and a hump-shaped subepithelial deposit located overlying the glomerular basement membrane reflection over the mesangium in a patient with C3 glomerulonephritis associated with monoclonal gammopathy (original magnification ×9,300). **k** | ‘Sausage-like’ thickening of the glomerular basement membrane associated with highly electron-dense intramembranous deposits in a patient with dense deposit disease associated with monoclonal gammopathy (original magnification ×4,800). MGRS, monoclonal gammopathy of renal significance.

Organized deposits of monoclonal immunoglobulins can be further divided into fibrillar, microtubular or crystalline and/or inclusionary forms (Fig. 3). Immunoglobulin-related amyloidosis, which includes subtypes with light-chain, heavy-chain and both heavy-and-light-chain deposition (AL, AH and AHL, respectively), has traditionally been recognized as the only condition in the fibrillar category^43^. However, monoclonal fibrillary glomerulonephritis has occasionally also been reported^44^. Amyloid fibrils stain with Congo red and are solid, non-branching and randomly arranged, with diameters of 7–12 nm (Fig. 3a). Amyloid fibrils involve glomeruli and blood vessels in the vast majority of patients and the interstitium in roughly 60% of patients (Fig. 2). Intratubular cytoplasmic AL amyloidosis occurs rarely^45^. The randomly arranged fibrils seen in fibrillary glomerulonephritis are on average twice as thick (10–30 nm) as those observed in amyloidosis (Fig. 3b) and generally do not stain with Congo red^44^. A small subgroup (7–17%) of patients with fibrillary glomerulonephritis demonstrates clinical evidence of a monoclonal gammopathy. In 3–15% of these patients, the IgG deposits exhibit light-chain restriction^44,46,47^, and this pathology is termed monoclonal fibrillary glomerulonephritis. Glomerular staining for DnaJ homologue subfamily B member 9 (DNAJB9) is a reliable marker for fibrillary glomerulonephritis^48^. This feature can be used to distinguish monoclonal fibrillary glomerulonephritis from AHL and AH amyloidosis, especially as fibrillary glomerulonephritis can sometimes show Congo red staining^49,50^.

Immunotactoid glomerulonephritis and cryoglobulinaemic glomerulonephritis are the two diseases that feature microtubular immunoglobulin deposits (Fig. 3c). Microtubules can be distinguished from fibrils by their hollow centres and large diameters (17–52 nm)^51^. Only type I and II cryoglobulinaemias are considered to be MGRS-associated disorders because type III cryoglobulinaemia is associated solely with polyclonal immunoglobulins. Immunotactoid glomerulonephritis is usually a renal-limited disease, whereas systemic manifestations including vasculitic rashes, peripheral neuropathy and arthralgias are common in patients with cryoglobulinaemia. Moreover, immunotactoid glomerulonephritis is not associated with cryoglobulinaemia and does not display the typical characteristics of cryoglobulinaemic glomerulonephritis (namely, glomerular protein thrombi and arterial or arteriolar vasculitic lesions). The glomerular deposits in immunotactoid glomerulonephritis are uniformly composed of microtubules, typically arranged in parallel arrays, with predominantly subepithelial and subendothelial localization. By contrast, only some of the deposits in cryoglobulinaemic glomerulonephritis are organized, and they usually appear as short, curved or straight microtubules (Fig. 3d) with predominantly intraluminal and subendothelial localization^52–54^. Of note, organized deposits are not always observed in cryoglobulinaemic glomerulonephritis^55^.

The crystalline and/or inclusions category consists of LCPT, crystal-storing histiocytosis (CSH) and (cryo)crystalglobulinaemic glomerulonephritis^56,57^. LCPT occurs as crystalline and non-crystalline variants. In the crystalline variant, numerous light-chain crystals of various shapes are seen within proximal tubular cells, inside lysosomes or freely in the cytoplasm (Figs 1, 3e). This variant is associated with κ light-chain deposition and complete or partial Fanconi syndrome^56–59^. In the non-crystalline variant, proximal tubular cells are distended and injured by the accumulation of numerous non-crystalline light-chain inclusions within lysosomes. This variant is typically associated with λ rather than κ light-chain deposition, and Fanconi syndrome is uncommon^56–59^. Rarely, non-crystalline LCPT can mimic acute tubular necrosis or acute interstitial nephritis^57,59^. In patients with CSH, light-chain crystals are often seen in renal histiocytes as well as in proximal tubular cells^60^ (Fig. 3f) and can have a widespread extrarenal distribution, including in bone marrow, lymph nodes, lungs, thyroid, parotid gland, cornea, synovium, skin, subcutaneous fat, stomach, liver and brain^61–64^. Finally, (cryo)crystalglobulinaemic glomerulonephritis is a rare monoclonal gammopathy characterized by immunoglobulin thrombi in the arterioles and glomerular capillaries. These thrombi exhibit a crystalline structure or periodicity on electron microscopy^65^. In some patients, the crystallization process in the periphery is precipitated by cold exposure, termed cryocrystalglobulinaemia^66^. Mesangial and endocapillary hypercellularity is often absent^65,66^. As in cryoglobulinaemia, intravascular crystal deposition results in small-vessel occlusion, thrombosis and/or inflammatory vasculitis^65,67^. Renal biopsy samples in patients with (cryo)crystalglobulinaemia reveal large extracellular crystals within glomerular capillaries and arterioles, which are frequently associated with fibrin thrombi and inflammation. Intracellular crystals can also be seen in patients with cryocrystalglobulinaemia (Fig. 3g).

### Lesions with non-organized deposits

Non-organized monoclonal immunoglobulin deposits are seen in patients with MIDD and those with proliferative glomerulonephritis with monoclonal immunoglobulin deposits (PGNMID). MIDD comprises a group of diseases characterized by deposition of light chains, heavy chains or both light and heavy chains^68–70^. In MIDD (Figs 1, 3h), linear punctate deposits are seen along both the glomerular basement membrane (GBM) and the tubular basement membrane (and occasionally extrarenally). By contrast, in PGNMID, deposits are confined to the glomeruli, where they are present in the mesangium and subendothelial space and occasionally in the subepithelial space (Figs 1, 3i). In addition, the deposits seen in PGNMID contain only intact immunoglobulins^24,71^, whereas those seen in heavy-chain MIDD or light-and-heavy-chain MIDD typically lack the first constant domain of the immunoglobulin^24,70,71^. In most patients, PGNMID is IgG3-driven, whereas truncated IgG1 is the most frequent immunoglobulin deposited in heavy-chain MIDD^70,72^. However, PGNMID can also be IgA-driven or (rarely) IgM-driven^72,73^.

### Lesions without deposits

Not all MGRS-associated renal lesions include monoclonal immunoglobulin deposits. The best example of an MGRS-associated disorder lacking such deposits is C3 glomerulopathy with monoclonal gammopathy, which includes both C3 glomerulonephritis and the rare entity of dense deposit disease. By definition, substantial renal immunoglobulin deposits will be absent in patients with C3 glomerulopathy, although 60–80% of patients aged >50 years with C3 glomerulopathy have a monoclonal gammopathy at the time of diagnosis^74–76^. This proportion far exceeds the expected rate in the general population^75–77^. Thus, although renal disease related to the monoclonal immunoglobulin can be demonstrated in only about 30% of patients affected by C3 glomerulopathy (in whom the monoclonal immunoglobulin acts as a C3 nephritic factor or anti-factor-H antibody), it should still be considered an MGRS-associated disorder^25,75^.

C3 glomerulonephritis and dense deposit disease are distinguished by their ultrastructural appearance: ill-defined, moderately electron-dense mesangial, subepithelial and subendothelial deposits are seen in C3 glomerulonephritis (Fig. 3j), whereas highly electron-dense ‘sausage-like’ intramembranous deposits and mesangial rounded nodular deposits are seen in dense deposit disease (Fig. 3k). Large ‘hump-shaped’ subepithelial deposits might be seen in either lesion^25^ (Fig. 3j). C3 glomerulonephritis is the most common form of C3 glomerulopathy with monoclonal gammopathy. Importantly, roughly 5–10% of patients with monoclonal gammopathy and findings on standard immunofluorescence (that is, conducted on frozen tissue) consistent with C3 glomerulonephritis will actually have a membranoproliferative glomerulonephritis with masked monoclonal deposits. These patients require additional immunofluorescence studies to be performed on protease-digested, paraffin-embedded tissue for identification of the monoclonal immunoglobulin in the deposits^78,79^.

### Lesions with provisional status

Thrombotic microangiopathy is the endothelial injury seen most commonly in microangiopathy with haemolytic anaemia (MAHA). Thrombotic microangiopathy and MAHA can occur concurrently in patients with monoclonal gammopathies, including MM and WM^13,42,80,81^. The pathophysiology of these disorders is not entirely understood but might be related to the monoclonal immunoglobulin acting as an autoantibody against a complement regulatory protein^82^. The other lesion in this category is glomerular microangiopathy associated with polyneuropathy, organomegaly, endocrinopathy, monoclonal gammopathy and skin changes (POEMS) syndrome^83,84^. The glomerular microangiopathy seen in POEMS syndrome is associated with a monoclonal gammopathy, which is nearly always λ light-chain type. However, the λ light chain itself is usually absent from kidney biopsy samples. Instead, the lesion is a subacute to chronic glomerular thrombotic microangiopathy characterized by mesangial and endothelial cell proliferation, mesangiolysis, widening of the subendothelial zone and double contouring^85^. Interestingly, these patients show no evidence of MAHA. The renal lesions in POEMS syndrome are thought to be secondary to a cytokine-mediated endothelial cell injury, similar to that seen in myeloproliferative neoplasm-related glomerulopathy^86^.

### Lesions classed as miscellaneous

The ‘miscellaneous’ subcategory of MGRS-associated lesions includes kidney diseases that are typically not associated with MGRS, such as anti-GBM disease secondary to a monoclonal gammopathy. The anti-GBM monoclonal antibody can be IgG or IgA^87–89^. In most patients with this disease, the anti-GBM antibody is not detectable in serum by commercially available enzyme-linked immunosorbent assay (ELISA) or multiplex flow immunoassays, which are designed to detect antibodies against only α3NC1. These patients experience frequent relapses and the disease recurs after kidney transplantation, which is not typical in patients with non-MGRS-associated anti-GBM disease^87–89^. A pattern of membranous nephropathy that is visually indistinguishable from that associated with polyclonal immunoglobulin-mediated membranous lesions on light microscopy and electron microscopy has been described in patients with monoclonal IgG deposits^90,91^. Although the phospholipase A2 receptor (PLA2R) was identified as the target of the monoclonal IgG in a single patient included in a small study, a larger study found that only 26% of patients showed evidence of antibodies to PLA2R and that none of those patients had a lymphoproliferative disorder^90,91^. Finally, Henoch–Schönlein purpura with IgA nephropathy has very occasionally been reported in patients with IgA monoclonal gammopathy or MM^92,93^.

## Evaluation of suspected MGRS

Owing to differences in clinical characteristics and therapy, it is essential to distinguish MGRS-associated disorders from kidney diseases that are unrelated to monoclonal immunoglobins^10,12,24,28,94,95^. In patients suspected of having MGRS, the evaluation starts with a kidney biopsy. If analysis of the biopsy sample identifies an MGRS-associated lesion, a haematological evaluation (including monoclonal immunoglobulin studies, clonal determination and cytogenetic analysis) should be performed. These steps are discussed in greater detail below.

### When to perform a renal biopsy

As MGRS is a haematological condition defined by its renal manifestations, a kidney biopsy is essential for its diagnosis. However, not every patient with a monoclonal gammopathy and kidney disease has MGRS. The frequency of MGUS is 3% in people aged >50 years, 5% in persons aged >70 years and as high as 8% in men aged >80 years^77^. The prevalence of MGUS is two to three times higher in African Americans than in white individuals of the same population^96^. The incidence of chronic kidney disease (CKD) also increases after age 60 years^97^. Therefore, the same patient could have both MGUS and CKD that are unrelated to each other. Studies from the same county in the United States found that the annual incidence of glomerular disease was approximately 1 per 100,000 individuals in the general population and that the prevalence of MGUS in people aged >50 years was 3.2%^77,98^. A renal biopsy study of patients with clinically suspected MGUS found that 45% of these patients did not have an MGRS-associated kidney disorder; however, as additional disease entities related to monoclonal gammopathies have been identified after the publication of this article, the true value might be lower^10^.

Clinicians must balance the risks associated with underdiagnosis of potentially treatable conditions against those of complications from the biopsy procedure. However, patients with MGRS-associated renal lesions (including amyloidosis) do not experience any increase in the risk of bleeding after kidney biopsy (which remains about 4%)^99,100^. Thus, performing a kidney biopsy in a patient with diabetes and rapidly progressive loss of renal function or increasing proteinuria is reasonable, especially if their diabetes is well controlled and/or evidence of extrarenal microvascular disease is absent. Because MGUS is uncommon in individuals aged <50 years (and is especially rare in those aged <40 years), its presence in people aged <50 years, when accompanied by renal manifestations, deserves a thorough evaluation. Older age (≥70 years) should not discourage biopsy as most MGRS-related renal diseases occur in patients aged >50 years. In young and physically fit patients who are eligible for kidney transplantation, a kidney biopsy should be performed provided the kidneys are not markedly shrunken. Transjugular kidney biopsy is an option in high-risk patients from whom it would otherwise be difficult to obtain kidney tissue^101,102^.

### Renal biopsy evaluation

**Fig. 4:**
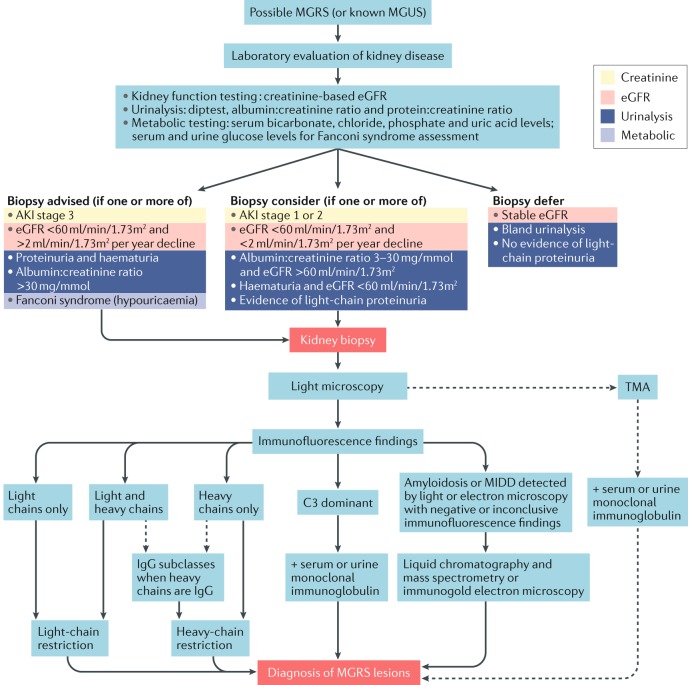
Algorithm for renal biopsy evaluation in patients suspected to have MGRS. Kidney biopsy analysis in patients suspected to have monoclonal gammopathy of renal significance (MGRS) should include light microscopy (including staining the paraffin sections with haematoxylin and eosin, periodic acid−Schiff, Masson trichrome, Jones methenamine silver and Congo red). Immunofluorescence studies conducted on frozen tissue should include staining for IgG, IgM, IgA, C1q, C3 and κ and λ light chains. Finally, transmission election microscopy should be conducted. This standard renal biopsy approach enables diagnosis of MGRS in the majority of affected patients. In some individuals, ancillary techniques are needed to establish the diagnosis, including mass spectrometry, immunogold electron microscopy, immunofluorescence staining for IgG subtypes and paraffin immunofluorescence. The indications for these ancillary techniques are detailed in Table 2. AKI, acute kidney injury; eGFR, estimated glomerular filtration rate; MGUS, monoclonal gammopathy of undetermined significance; MIDD, monoclonal immunoglobulin deposition disease; TMA, thrombotic microangiopathy.

The diagnosis of MGRS-associated lesions requires the integration of morphological alterations seen on light microscopy with the findings of immunohistochemistry (immunofluorescence or immunoperoxidase) and transmission electron microscopy studies, as well as correlation with the patient’s medical history and laboratory findings. In some patients, ancillary techniques are needed to establish the diagnosis, including protease immunofluorescence, ultrastructural immunogold labelling and laser microdissection followed by liquid chromatography and mass spectrometry (LC–MS). A detailed description of our consensus recommendations for renal biopsy and the indications for ancillary techniques is provided in Table 2. Our recommended approach to renal biopsy analysis in patients suspected to have MGRS is provided in Fig. 4.

**Table 2:** Consensus recommendations for the evaluation of MGRS-associated disorders

Modality	Recommendations	Refs
Kidney biopsy	Recommended in the following patients: • Those with monoclonal gammopathy and unexplained kidney disease • Those with known risk factors for chronic kidney disease but an atypical clinical course • Patients with kidney disease and monoclonal gammopathy aged <50 years	NA
Protease immunofluorescence on kidney biopsy	Recommended in the following scenarios: • When glomeruli are lacking in frozen tissue samples • In patients with suspected LCPT and other forms of crystalline nephropathies, such as CSH and crystalglobulin-induced nephropathy • In patients with a monoclonal gammopathy in whom kidney biopsy samples show C3 glomerulonephritis or unclassified proliferative glomerulonephritis in the context of negative findings by immunofluorescence on frozen tissue samples (including in patients with features of cryoglobulinaemic glomerulonephritis on light or electron microscopy) • In patients with fibrillary glomerulonephritis who have apparent light-chain restriction detected by immunofluorescence on frozen tissue	NA
Renal amyloid typing by liquid chromatography and mass spectrometry	Recommended in the following situations: • When frozen tissue for immunofluorescence is not available • Negative immunofluorescence staining for κ and λ light chains, with negative immunoperoxidase staining for SAA and LECT2 • Equal staining for κ and λ light chains by immunofluorescence • Bright staining for IgG and/or IgA by immunofluorescence • Equivocal Congo red staining • To enable distinction between AHL amyloidosis and congophilic fibrillary glomerulonephritis	108
Flow cytometry or other immunotyping	• Neoplastic plasma cells frequently show aberrant loss of CD45 and CD19, as well as aberrant expression of CD56 and CD117; therefore, these markers (in addition to κ and λ light chains and CD38) are useful in identifying small plasma cell clones • Including CD5 and CD20 in the immunophenotyping of B cells can frequently separate small clones from polytypic cells • The most sensitive assay available at a given institution should be used. Although there is no established gold standard, many laboratories have the capability to determine minimal residual disease in MGRS at a sensitivity of 10^−4^ to 10^−6^ monoclonal cells. The sensitivity of flow cytometry immunophenotyping depends on the total number of collected cells, the number of antibodies used to find an aberrant phenotype, the phenotype of the abnormal clone and sample quality	^118^
Immunohistochemistry	• Immunohistochemistry of bone marrow biopsy samples has a low sensitivity for detecting κ-expressing and λ-expressing plasma cells and could be useful only if there is a major plasma cell clone and a lack of polyclonal plasma cells • Immunohistochemistry might be useful in the evaluation of atypical lymphoid infiltrates, particularly if flow cytometry is not available or infiltrates are very focal • If an abnormal clone is detected, the light-chain isotype should be compared with that present in renal lesions and additional information should be obtained	NA
Mutational analysis	The MYD88 L265P mutation is found in over 90% of patients with lymphoplasmacytic lymphoma or Waldenström macroglobulinaemia but in only 40–60% of individuals with IgM MGUS	^119–121^
FISH	Cyclin D1 FISH with immunostaining for CD10, BCL2 and BCL6 to subclassify diffuse large cell lymphoma, and prognostic FISH panels for MM and CLL, can also be useful	^119–121^

AHL, immunoglobulin A heavy-and-light chain; CLL, chronic lymphocytic leukaemia; CSH, crystal-storing histiocytosis; FISH, fluorescence in situ hybridization; LCPT, light-chain proximal tubulopathy; LECT2, leukocyte cell-derived chemotaxin 2; MGRS, monoclonal gammopathy of renal significance; MGUS, monoclonal gammopathy of undetermined significance; MM, multiple myeloma; NA, not applicable; SAA, serum amyloid A protein.

To confirm the monotypic nature of the immunoglobulin deposits, immunofluorescence staining for IgG subclasses should be performed in biopsy samples from patients with glomerular disorders related to deposition of an intact monoclonal IgG (such as PGNMID, immunotactoid glomerulopathy, type I cryoglobulinaemic glomerulonephritis and monoclonal membranous nephropathy) or of a truncated monoclonal heavy chain (such as heavy-chain deposition disease, heavy-and-light-chain deposition disease or heavy-chain amyloidosis). Of note, IgG subclass restriction is not sufficient by itself to establish monoclonality, as some non-MGRS glomerular diseases — such as PLA2R-associated polyclonal membranous nephropathy and non-monoclonal fibrillary glomerulonephritis — commonly show staining restricted to one IgG subclass but positive staining for both κ and λ light chains^23,103^. Identification of complement C1q and/or C3 proteins within the monotypic renal deposits might reveal the cause of hypocomplementaemia in patients with MGRS-associated lesions such as PGNMID, immunotactoid glomerulonephritis, type I cryoglobulinaemic glomerulonephritis, C3 glomerulonephritis and heavy-chain or heavy-and-light-chain deposition disease^23,24,51,52,68,103,104^.

Electron microscopy is often necessary to identify the specific MGRS-associated lesion. Ideally, electron microscopy should be performed on 2–3% glutaraldehyde-fixed tissue. If glutaraldehyde-fixed tissue is not available or lacks glomeruli, formalin-fixed, paraffin-embedded tissue samples can be reprocessed for electron microscopy. Although reprocessing is usually associated with various artefacts, the immune deposits generally remain sufficiently intact to permit an accurate assessment of their location and structure. Electron microscopy of frozen tissue samples or tissue fixed in Zenker or B5 fixatives is not recommended owing to its generally very poor ultrastructural preservation. In patients with monoclonal gammopathy, at least two glomeruli should be studied ultrastructurally as glomerular deposits can be sparse and only a portion of the deposits show substructural features. For example, in most patients with cryoglobulinaemic glomerulonephritis, only a small number of deposits (most commonly intraluminal ones) exhibit the microtubular substructure that is so useful in establishing the diagnosis^53^. In patients with monoclonal gammopathy, a directed search for intratubular cytoplasmic crystals or inclusions by electron microscopy is of paramount importance, as these features can be overlooked by light microscopy and standard immunofluorescence studies on frozen tissue. Additionally, in some patients with classic MIDD, the characteristic punctate, powdery, electron-dense deposits can be found only in small sections of the tubular basement membranes. Therefore, a thorough search for tubular basement membrane deposits by electron microscopy is necessary to distinguish classic MIDD from MIDD identified by immunofluorescence only^69^. Ultrastructural immunogold labelling is a sensitive technique that can assist in the histopathological diagnosis of MGRS-associated lesions, such as AL amyloidosis, MIDD, LCPT and CSH, by confirming the location and composition of monoclonal deposits, but it is not widely available^35,60,70,105–107^.

Laser microdissection followed by LC–MS is currently the gold standard for amyloid typing but is available in only a few specialized centres. In renal pathology laboratories that routinely perform immunofluorescence studies on native kidney biopsy samples, LC–MS is essential for typing renal amyloidosis in about 15% of patients^43,108^. LC–MS is crucial for the diagnosis of rare hereditary forms of renal amyloidosis that cannot be typed by immunofluorescence, but it is also important to distinguish AH and AHL amyloidoses from non-immunoglobulin amyloidoses associated with nonspecifically entrapped immunoglobulins (particularly AA amyloidosis) and from fibrillary glomerulonephritis^49,109^. LC–MS can also be useful in the diagnosis of MGRS-associated lesions other than immunoglobulin amyloidosis when immunofluorescence studies are not available or have negative findings. An example of the latter situation is IgD heavy-chain deposition disease, which is generally missed by immunofluorescence studies because an IgD antibody is not included in the routine immunofluorescence panel^110^.

### Monoclonal immunoglobulin testing

Once the diagnosis of an MGRS-associated lesion has been established, a search for the culprit monoclonal immunoglobulin should be undertaken (if it has not been identified already). Protein electrophoresis analyses of serum and urine samples are the first tests performed^111^. Although its sensitivity is inferior to that of some other tests discussed here, serum protein electrophoresis is quantitative, easy to perform and inexpensive. Urine protein electrophoresis is less sensitive than serum protein electrophoresis but provides the total protein level, urinary albumin level and globular protein (monoclonal immunoglobulin or light chain) component — parameters that are necessary for diagnosis, prognostication and response assessment^112–114^. Immunofixation of a serum sample and of a concentrated urine aliquot from a 24 h collection should also be done because this test is more sensitive than protein electrophoresis. Immunofixation is necessary for the identification and typing of monoclonal immunoglobulins, as well as for the determination of a complete response^113,115^. Immunoblotting is a highly sensitive technique that can detect small amounts of monoclonal immunoglobulin, characterize the distribution of IgG heavy-chain subclasses and detect deletion of the first constant domain, the hallmark of heavy-chain deposition disease and AH amyloidosis^70^. However, this technique is not widely available.

Another critical test is the serum free light-chain assay, which detects unbound free light chains^113^. This assay measures κ and λ free light chains independently and can be used to determine the κ:λ free light-chain ratio. Clonality can be inferred from an abnormal κ:λ free light-chain ratio: a high ratio indicates a κ clone whereas a low ratio indicates a λ clone. Because free light chains are cleared by the kidney, impaired renal function alters the free light-chain concentration. The ‘normal’ free light-chain ratio, 0.26–1.65, can rise to 0.34–3.10 in patients with severe renal impairment (CKD stage 5 or greater), but small declines in renal function can also impair free light-chain clearance^116^. Knowing which serum free light-chain assay is being used by the laboratory is extremely important, as at least two major assays are currently on the market. Not only are the results of these assays mathematically inconvertible, but the effects of renal impairment differ between these assays; the evidence suggests that the N Latex assay is less affected than the FreeLite assay by impaired renal function^117^. Thus, the same assay must be used to monitor a particular patient throughout their treatment. Moreover, given that the two assays have different performance characteristics, free light-chain levels might need to be checked using the other assay if the first result is negative. In addition, serum immunofixation might be more helpful than serum free light-chain assays in diseases associated with an intact monoclonal immunoglobulin (such as PGNMID)^72^. Finally, although antibodies for use in urinary light-chain assays have been developed, these assays have not been validated and should not be used to quantify the amount of light chain (Bence Jones protein) in a 24 h urine specimen (which should instead be measured by urine protein electrophoresis, as previously stated)^118^.

Identification of the culprit monoclonal immunoglobulin has important diagnostic and prognostic consequences. The monoclonal immunoglobulin detected in serum and/or urine must match that found in immunoglobulin deposits in the kidney^35^; if the immunoglobulin found in renal deposits differs from that found in the circulation, the monoclonality of the putative culprit immunoglobulin is called into question. Although the serum M-spike concentration and serum free light-chain assay results have both diagnostic and prognostic importance, the correlation between the results of these tests and the severity or type of kidney disease is less well established.

## Clonal identification

The diagnosis of MGRS should generally be established before obtaining a haematological consultation. The focus of the haematologist and/or oncologist and haematopathologists should be clonal identification, which is central to the management of patients with MGRS. The only exception is when the patient has already been diagnosed as having MM, WM or CLL, which eliminates the need for a kidney biopsy (because treatment will be initiated regardless of the kidney lesions present). Clonal identification is essential because the same kidney diseases can occur in different haematological disorders (Table 3). Of note, although a pathological clone can be identified in virtually every patient with AL amyloidosis or MIDD, such clones are often difficult to detect in other diseases. For example, the chance of identifying the pathological clone falls below 17% for patients who do not have a detectable monoclonal immunoglobulin on immunofixation studies^72^, and only 20–30% of patients with PGNMID have a detectable circulating monoclonal immunoglobulin^24,72^. As treatment differs according to whether the clone has a plasmacytic or lymphocytic nature, choosing the right agent is challenging if a clone cannot be identified.

**Table 3:** Renal lesions associated with monoclonal gammopathy

Lesion	Proportion of lesions (%)					
	Monoclonal immunoglobulin deposits	Detectable monoclonal immunoglobulin	MM	MGRS	Other^a^	Refs
Light-chain cast nephropathy	100	100	99	0	~1	^2,4,11,13^
Immunoglobulin-related amyloid amyloidosis	96	99	16	80	1–4	^43,113,128,129^
MIDD	100	100	0–20	78–100	1–2	^29,31,68,130,131^
Light-chain proximal tubulopathy	100	97^b^	12–33	61–80	3–8	^32,56,58,132^
Cryoglobulinaemic (type I) glomerulonephritis	100	90–100	6–8	47–52	24–56	^133–136^
Cryoglobulinaemic (type II) glomerulonephritis	100	49	0	20	7	^133–136^
PGNMID	100	30–32	4	96	~1	^24,72^
Crystal-storing histiocytosis	83	90	33	8	50	^137^
Cryocrystalglobulin or crystalglobulin nephropathy	91	82	61	18	4	^138^
Immunotactoid glomerulonephritis	69–93	63–71	0–13	25–50	25–50	^23,51^
C3 glomerulopathy with monoclonal gammopathy^c^	0	28–83^d^	0–40^d^	40–90	6–10	^25,74,75,104^
Monoclonal fibrillary glomerulonephritis^e^	100	7–17	0–54	55–98	2–10	^44,47,139^

MGRS, monoclonal gammopathy of renal significance; MIDD, monoclonal immunoglobulin deposition disease; MM, multiple myeloma; PGNMID, proliferative glomerulonephritis with monoclonal immunoglobulin deposits. ^a^Haematological conditions including lymphoplasmacytic lymphoma (Waldenström macroglobulinaemia), smouldering Waldenström macroglobulinaemia, B cell lymphomas, chronic lymphocytic lymphoma and monoclonal B cell lymphocytosis. ^b^Sensitivity increased by immunofluorescence after pronase digestion. ^c^Most instances of fibrillary glomerulonephritis and C3 glomerulopathy are not associated with a monoclonal gammopathy. The percentages for MM, MGRS and other haematological conditions relate to the group of patients who do have a monoclonal gammopathy. ^d^Patients over the age of 50 years. ^e^In these patients, the glomerular deposits show light-chain restriction or stain for IgG without light chains, both by frozen tissue and paraffin tissue immunofluorescence (as in 15–17% of patients with fibrillary glomerulonephritis).

Bone marrow aspiration and biopsy should be performed to evaluate MGRS in most patients, although in patients with CLL clones, the diagnosis could be made with peripheral blood flow cytometry. Morphological assessment should include quantification of the percentage of plasma cells (in plasma cell clones) and evaluation for the presence of atypical lymphoid or lymphoplasmacytic aggregates (in lymphoma clones) as well as amyloid deposits. In addition, ancillary studies — in particular, flow cytometry immunophenotyping, detection of minimal residual disease and cytogenetic and genetic evaluation of the clones — are helpful for the identification of small clones as well as for deriving treatment recommendations^119–121^. The myeloma fluorescent in situ hybridization (FISH) panel has shown increasing importance in guiding the treatment of patients with plasma cell dyscrasias. For example, patients with AL amyloidosis featuring translocation t(11;14) have inferior responses to bortezomib-based therapy, whereas those with gain of chromosome 1q21 show poorer responses to melphalan plus dexamethasone^99,100,122–124^ (versus patients without these genetic variants). These findings highlight the importance of performing the myeloma FISH panel on all bone marrow biopsy samples from patients with plasma cell dyscrasia.

If bone marrow evaluation does not reveal a clonal haematological disorder, the next step could be to perform imaging studies (such as CT with or without PET, or whole-body MRI) to look for a localized plasmacytoma or for lymphadenopathy in low-stage, low-grade lymphoma^34,35^. For patients suspected to have MM, whole-body CT with or without PET (or MRI) should be performed to look for bone disease^125,126^. Any suspicious lesions should be biopsied and enough material should be obtained to enable diagnostic and prognostic studies. Next-generation flow cytometry has been used in the measurement of minimal residual disease^127^. This technique might be helpful in patients suspected of having MGRS who have negative findings on traditional cytology or flow cytometry studies of bone marrow samples.

## Summary

MGRS is a new classification of pathogenic clonal proliferative disorders that produce a nephrotoxic protein. The term MGRS was needed to improve the classification of these diseases for research purposes, and to accurately categorize them as pathological, so that government agencies could allocate the resources necessary for their treatment. The diagnosis of MGRS can be established only by performing a kidney biopsy that either demonstrates the presence of monotypic immunoglobulin deposits or infers their involvement in the case of C3 glomerulonephritis or thrombotic microangiopathy with a circulating monoclonal immunoglobulin. Clinicians will need to balance the risk of missing a diagnosis against those of the complications of renal biopsy; therefore, the judicious use of renal biopsy is important. Detection of a monoclonal immunoglobulin, in addition to helping to establish the diagnosis of MGRS, has diagnostic and prognostic value and is also used to predict treatment responses. Haematological evaluation might require peripheral blood flow cytometry, bone marrow biopsy and imaging studies to assess localized disease.

## Glossary

Bence Jones protein: Monoclonal immunoglobulin light chains detected in the urine of patients with multiple myeloma and other haematological malignancies.
Dangerous small B cell clones: Size is not everything — even small B cell-derived clones might synthesize a very toxic monoclonal immunoglobulin that produces devastating systemic damage and a progressive or even fatal clinical course.
